# Heritability of Intraindividual Mean and Variability of Positive and Negative Affect: Genetic Analysis of Daily Affect Ratings Over a Month

**DOI:** 10.1177/0956797616669994

**Published:** 2016-10-11

**Authors:** Yao Zheng, Robert Plomin, Sophie von Stumm

**Affiliations:** 1 Department of Psychology, Simon Fraser University; 2 Child & Family Research Institute, Vancouver, British Columbia, Canada; 3 Medical Research Council Social, Genetic & Developmental Psychiatry Centre, Institute of Psychiatry, Psychology & Neuroscience, King’s College London; 4 Department of Psychology, Goldsmiths, University of London

**Keywords:** positive and negative affect, heritability, daily diary, two-factor theory, twin study

## Abstract

Positive affect (e.g., attentiveness) and negative affect (e.g., upset) fluctuate over time. We examined genetic influences on interindividual differences in the day-to-day variability of affect (i.e., ups and downs) and in average affect over the duration of a month. Once a day, 17-year-old twins in the United Kingdom (*N* = 447) rated their positive and negative affect online. The mean and standard deviation of each individual’s daily ratings across the month were used as the measures of that individual’s average affect and variability of affect. Analyses revealed that the average of negative affect was significantly heritable (.53), but the average of positive affect was not; instead, the latter showed significant shared environmental influences (.42). Fluctuations across the month were significantly heritable for both negative affect (.54) and positive affect (.34). The findings support the two-factor theory of affect, which posits that positive affect is more situational and negative affect is more dispositional.

Positive and negative affect refer to the extent to which a person experiences emotional states such as enthusiasm or alertness and distress or unhappiness, respectively (Diener & Emmons, 1984; Diener, Larsen, Levine, & Emmons, 1985; Watson, 1988). Although they both inform a wide range of physical- and mental-health outcomes (Diener, 2000; Watson, 1988), positive and negative affect are relatively independent dimensions and correlate with different personality traits (Diener & Emmons, 1984; Diener et al., 1985; Emmons & Diener, 1985; Watson, 1988). They may also differ etiologically. According to the two-factor theory of affect (Diener & Larsen, 1984; Emmons & Diener, 1985), positive affect has primarily a situational etiology and is influenced more by environmental experiences, whereas negative affect has a dispositional etiology and is influenced more by personality traits.

The twin study design enables a genetic test of the principal hypothesis derived from the two-factor theory: that interindividual differences in positive affect are more environmentally driven, and interindividual differences in negative affect are more genetically driven. Consistent with this hypothesis, a study of 105 twin pairs (18–72 years old) found moderate genetic influences on negative affect (.34) but not on positive affect (.00) when differences in affect were assessed as traits at one time point (Baker, Cesa, Gatz, & Mellins, 1992). Another study, with 300 pairs of 18- to 70-year-old twins, found similar results for genetic influences on negative and positive affect assessed as traits at one time point (.24 vs. .18; Riemann, Angleitner, Borkenau, & Eid, 1998). Also, in a study of 260 pairs of 18- to 46-year-old female twins, genetic influences on the average of positive affect assessed over 5 consecutive days were nonsignificant (.19; Menne-Lothmann et al., 2012).

In addition to showing interindividual differences as a trait, affect fluctuates within individuals over time, and this intraindividual variability contains unique information beyond that provided by average affect (Nesselroade & Ram, 2004; Ram & Gerstorf, 2009; Wang, Hamaker, & Bergeman, 2012; Watson, 1988), especially for predicting physical- and mental-health outcomes (Clark & Watson, 1988; Eid & Diener, 1999; Watson, 1988). For instance, higher levels of intraindividual variability in affect are salient markers for poorer physical and mental health in children and adolescents (Shomaker & Reina, 2015).

Notwithstanding their predictive validity, the etiology of fluctuations in affect is undetermined to date. In a study of 279 pairs of 18- to 46-year-old female twins assessed 10 times per day over the course of 5 consecutive days, genetic influences accounted for .35 of the intraindividual variability in negative affect and .18 of the intraindividual variability in positive affect (Jacobs et al., 2013). By contrast, in a sample of 210 twin pairs ages 25 through 74 years assessed once per day over the course of 8 consecutive days, the genetic influences on intraindividual variability of negative affect were negligible (.00; Neiss & Almeida, 2004). These inconsistencies in findings are likely due to the different frequencies and durations of assessments in the studies, because the reliability of day-to-day intraindividual variability of affect, as well as average affect, is very low when the assessment period is less than 2 weeks long (Estabrook, Grimm, & Bowles, 2012; Wang & Grimm, 2012). Approximately a month’s assessment is needed to reach satisfactory reliability (> .91) for day-to-day intraindividual variability in affect and average affect (Estabrook et al., 2012; Wang & Grimm, 2012). Another likely reason for the findings’ discrepancy is the difference in ages between the two tested samples, because average negative affect may be more heritable in younger people than in older people (Neiss & Almeida, 2004).

The twin study design distinguishes shared environmental influences that make family members similar (nurture) from nonshared environmental influences that differentiate members of the same family (Plomin, 2011). Studies consistent with the hypothesis derived from the two-factor theory have demonstrated substantial shared environmental influences for traitlike positive affect (i.e., single assessment; .31; Baker et al., 1992) and for the average of positive affect across repeated assessments (.34; Menne-Lothmann et al., 2012). However, other studies have produced lower estimates for shared environmental influences on the intraindividual variability of positive and negative affect (.00 in Jacobs et al., 2013; .15 in Neiss & Almeida, 2004). The discrepancy in these latter results for intraindividual variability is again likely due to differences in the frequency with which affect was assessed and in the samples’ age ranges.

In the current study, we examined genetic influences on the average of and on the day-to-day variability in positive and negative affect. A subsample of 17-year-old twins from the Twins Early Development Study (TEDS; Haworth, Davis, & Plomin, 2013) were assessed daily on their positive and negative affect over the course of 40 days using a customized online Web application. Following previous twin studies examining daily affect (Jacobs et al., 2013; Menne-Lothmann et al., 2012; Neiss & Almeida, 2004), we used the mean and standard deviation of each participant’s daily ratings over the 40 days as the measures of that person’s mean affect and variability of affect, respectively. On the basis of the two-factor theory of affect, which posits that positive affect is more situational and negative affect is more dispositional, we predicted that both average negative affect and intraindividual variability in negative affect would be significantly heritable, whereas average positive affect and intraindividual variability in positive affect would show significant shared environmental influences.

## Method

### Participants and procedure

TEDS is a longitudinal study of twins born in England and Wales in 1994, 1995, and 1996. Detailed descriptions of the recruitment procedure and the sample are provided elsewhere (Haworth et al., 2013). Zygosity was assessed with a parental questionnaire that has been shown to be more than 95% accurate, using direct genetic testing as the benchmark (Price et al., 2000). DNA testing was conducted when zygosity was unclear. The institutional review board at King’s College London approved the procedure. Informed consent for the present study was obtained from both parents and twins before data collection.

A total of 314 parents of 17-year-old same-sex twins (610 invited families, 51.5% consent rate) agreed for their children to participate in a study of daily mood that would last 40 consecutive days. The demographics of the families consenting to participate (93.0% White; socioeconomic status, standardized SES = 0.30, *SD* = 0.96) were similar to those of the entire group of invited families (91.3% White; standardized SES = 0.23, *SD* = 0.97) and the total TEDS sample (91.7% White; standardized SES = 0.00, *SD* = 1.00), but with a somewhat lower participation rate for males (43.3% vs. 50.1% vs. 49.9%).

The twins were instructed to use an online application every day between 3 p.m. (i.e., after school) and 2 a.m. that night to fill out a brief survey about their general mood that day. Each participant filled out the questionnaire at roughly the same time each day (intraindividual standard deviation: *M* = 1.7 hr, *SD* = 0.6 hr, range = 0.1–3.2 hr). A total of 275 pairs of twins and 3 unpaired twins (*n* = 553; 41.4% males; 93.9% White; standardized SES = 0.31, *SD* = 0.96) submitted daily reports within the designated 1-week time window for starting this study. This group included 121 monozygotic (MZ) twin pairs, 154 dizygotic (DZ) twin pairs, and 3 unpaired DZ twins. On average, each twin provided 33.7 daily reports (range = 1–40, *SD* = 8.8), and 87.2% of the twins provided valid reports on at least 30 days. Participants with major medical problems or severe perinatal problems were excluded from the analyses, as were those whose first language was not English. Because the reliability of intraindividual means and standard deviations is generally lower with fewer assessments (Estabrook et al., 2012; Wang & Grimm, 2012), only data from twins who filled out the survey on 30 or more days were used in the analyses reported here. The final sample consisted of 447 twins (96 MZ and 122 DZ twin pairs, 11 unpaired twins), who as a group completed more than 15,000 assessments.

### Measures

Daily mood was measured with the short form of the Positive and Negative Affect Schedule (PANAS; Watson, Clark, & Tellegen, 1988), which has been widely used in studies assessing mood and affect, including a study of a nonclinical British sample representative of the general population (Crawford & Henry, 2004). The PANAS taps into the intensity of approach and withdrawal motivation (Harmon-Jones, Harmon-Jones, Abramson, & Peterson, 2009). The 10-item short-form PANAS has shown satisfactory internal consistency and test-retest reliability in multiple countries, including the United Kingdom, and it has good convergent validity with measures of subjective well-being and subjective happiness (Thompson, 2007). The rating procedure followed the end-of-day daily-diary design. Participants were instructed to consider how they had felt over the course of the whole day, rather than just at the time of their response, and to rate the extent to which they had felt each of 10 affect states, using a 5-point scale from 1, *very slightly or not at all*, to 5, *extremely*. Five items—*active, alert, attentive, determined*, and *inspired*—assessed positive affect, and five items—*afraid, ashamed, hostile, nervous*, and *upset*—assessed negative affect. The order of the items was altered randomly each day. The internal consistency (Cronbach’s α) ranged from .75 to .84 across days for positive affect and from .76 to .85 across days for negative affect. For positive affect, within-person reliability was .74, and between-person reliability was .80. For negative affect, within-person reliability (Cranford et al., 2006) was .67, and between-person reliability was .77.

Daily average scores were calculated separately for positive and negative affect. Systematic linear trend and weekly cyclic mean trend were removed from each individual time series (Ram & Gerstorf, 2009; Wang et al., 2012). The mean and standard deviation of each participant’s daily averages for positive and negative affect, across all assessments, were used as the measures of intraindividual mean affect and intraindividual variability of affect, respectively. All measures were normally distributed except for intraindividual mean of negative affect, which was positively skewed (initial skewness = 2.16) and thus logarithm-transformed (skewness = 1.01). All measures were corrected for mean effects of sex, and the residuals obtained after this correction were used in all subsequent analyses (McGue & Bouchard, 1984).

### Analyses

Standard twin model fitting was used to conduct univariate analyses of genetic and environmental influences on the variance of intraindividual means and standard deviations of positive and negative affect. Twin analyses make use of the difference in genetic resemblance between MZ twins, who share all of their segregating genes, and DZ twins, who share on average half of their segregating genes. In the standard twin model, called *ACE*, phenotypic variance is decomposed into three independent components: additive genetic influences (*A*) that make twins similar to each other, shared environmental influences (*C*) that make twins similar to each other, and nonshared environmental influences (*E*) that make twins different from each other; *E* also includes measurement error. The between-twin correlation for *A* is 1 for MZ twins and .5 for DZ twins, a reflection of their genetic resemblance. The between-twin correlation for *C* is 1 for both MZ and DZ twins growing up in the same household. Nonshared environmental influences are not correlated between twins (Plomin, DeFries, Knopik, & Neiderhiser, 2013).

Higher intraclass correlations for MZ twins than for DZ twins suggest additive genetic influences (*A*) and shared environmental influences (*C*). Shared environmental influences can be inferred from the remaining familial resemblance not explained by additive genetic influences and can be estimated by subtracting estimated *A* from the correlation for MZ twins. Nonshared environmental influences and measurement error (*E*) can be inferred by the extent to which the correlation for MZ twins is less than 1. To the extent that the correlation for MZ twins is more than twice that for DZ twins, nonadditive genetic influences (dominance, *D*) are suggested; *D* models the interactions of alleles at the same locus or on different loci (epistasis). The between-twin correlation for *D* is 1 for MZ twins and .25 for DZ twins (Plomin et al., 2013). In our analyses, when dominant genetic influences were suggested by the intraclass correlation, we also fit an *ADE* model.

All our twin analyses were conducted using OpenMx, a package for structural equation modeling; raw-data maximum likelihood estimation was used to handle missing data (Boker et al., 2011). We report the resulting parameter estimates, 95% confidence intervals (CIs), and model-fit statistics. OpenMx assesses model goodness of fit using −2 times the log likelihood (−2LL). To compare the fit of a full model with the fits of nested submodels (reduced models with fewer parameters), we used chi-square tests, with the degrees of freedom equal to the difference between the models in the number of parameters estimated. A nonsignificant chi-square test suggests that the reduced model is more parsimonious. We also computed Akaike’s information criterion (AIC). A smaller value of AIC suggests a better fit.

## Results

Table 1 provides descriptive statistics for the intraindividual means and variability of positive and negative affect. Participants generally rated their positive affect as moderate, but there was substantial interindividual variation (*M* = 2.80, *SD* = 0.61). Their average ratings of their negative affect were lower (indicating no or slight feelings of negative affect), but again, there was substantial variation between participants (*M* = 1.50, *SD* = 0.47). On average, individuals in the sample also experienced intraindividual variability of positive and negative affect (*M*s = 0.48 and 0.34, respectively), which suggests that their mood went up and down substantially over time. However, there were also interindividual differences in how much positive and negative mood fluctuated over the course of the study (*SD*s = 0.15 and 0.19, respectively), which suggests that some individuals experienced more ups and downs than others.

**Table 1.** Descriptive Statistics for Intraindividual Means and Standard Deviations of Positive and Negative Affect

Measure	*M*	*SD*	Skewness	Range
Positive affect				
Intraindividual mean	2.80	0.61	0.08	1.16–4.98
Intraindividual standard deviation	0.48	0.15	0.60	0.09–1.10
Negative affect				
Intraindividual mean	1.50	0.47	2.16	1.00–4.65
Intraindividual standard deviation	0.34	0.19	0.78	0.00–1.20

### Analyses of phenotypic correlations

Phenotypic correlations were calculated using a fully independent sample by randomly selecting 1 twin per pair (see Table S1 in the Supplemental Material). Intraindividual means of monthlong positive and negative affect were not correlated with each other (*r* = .00), which corroborated previous findings that positive and negative affect are independent dimensions.^1^ Similarly, intraindividual mean positive affect and intraindividual variability of positive affect were not intercorrelated (*r* = .08), which suggests that they are independent dimensions of individual differences. In contrast, intraindividual mean negative affect and intraindividual variability of negative affect were correlated with each other (*r* = .80, *p* < .001). Despite the substantial correlation, about 36% (1 – *r*^2^) of the variance in intraindividual mean negative affect was independent from intraindividual variability of negative affect. Also, because the raw intraindividual means of negative affect were positively skewed and most participants reported low levels of negative affect, the correlation between intraindividual mean negative affect and intraindividual variability of negative affect was likely due to floor effects (i.e., individuals with higher average negative affect had more room to fluctuate over time). Finally, intraindividual variabilities of positive and negative affect were moderately correlated (*r* = .43, *p* < .001); those individuals in the sample who fluctuated more in their daily positive affect also tended to fluctuate more in their daily negative affect. We repeated the analyses using the other co-twin in each pair, and the results were virtually identical (see Table S2 in the Supplemental Material).

### Univariate genetic analyses

**Fig. 1.**
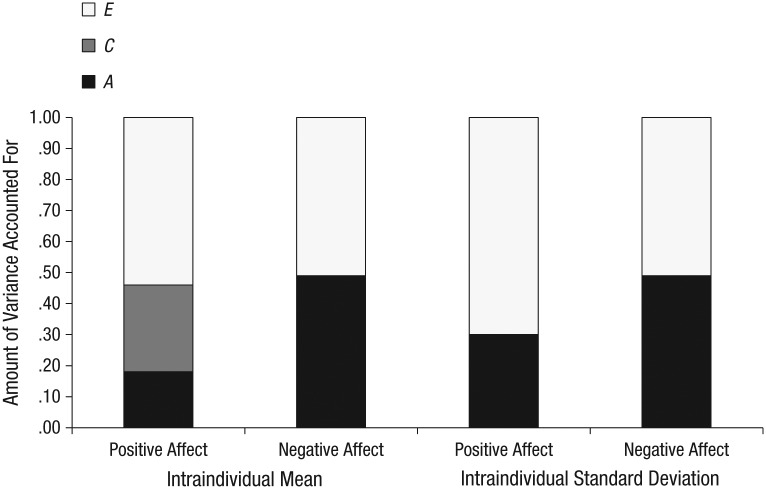
Results of the univariate genetic analyses of the intraindividual average of positive and negative affect and the intraindividual variability of positive and negative affect over the course of 1 month. For each measure, the graph shows the amount of variance due to additive-genetic influences (*A*), shared environmental influences (*C*), and nonshared environmental influences (*E*; including measurement error).

Table 2 presents the intraclass correlations for MZ and DZ twins. Across all four measures, the correlations for MZ twins were consistently higher than those for DZ twins, which suggests genetic influences. Tables 3 and 4 summarize the results for all the univariate models (full and reduced models) tested. Results for the classical *ACE* model (see Fig. 1, Tables 3 and 4) showed that average negative affect across the month was significantly heritable (.49, 95% CI = [.20, .62]), but average positive affect was not (.18, 95% CI = [.00, .56]). The intraindividual variability of negative affect across the month was significantly heritable (.50, 95% CI = [.28, .62]), as was the intraindividual variability of positive affect (.30, 95% CI = [.06, .44]).

**Table 2.** Intraclass Twin Correlations for Intraindividual Means and Standard Deviations of Positive and Negative Affect

	Positive affect		Negative affect	
Zygosity group	Intraindividual means	Intraindividual standard deviations	Intraindividual means	Intraindividual standard deviations
Monozygotic twins	.46 [.31, .58]	.35 [.19, .49]	.50 [.32, .63]	.55 [.41, .65]
Dizygotic twins	.37 [.19, .52]	–.02 [–.22, .17]	.17 [.003, .33]	.10 [–.10, .28]

Note: Values in brackets are 95% confidence intervals.

**Table 3.** Univariate Model-Fitting Results and Fit Statistics for Intraindividual Means of Positive and Negative Affect

			Results of model comparison					
Model	–2LL	AIC	Full model compared	Δχ^2^	*p*	*A*	*C* or *D*	*E*
Intraindividual means of positive affect								
*ACE*	770.87	–115.13	—	—	—	0.18 [0.00, 0.56]	0.28 [0.00, 0.51]	0.54 [0.42, 0.68]
*ADE*	773.16	–112.84	—	—	—	0.49 [0.10, 0.60]	0.00 [0.00, 0.39]	0.51 [0.40, 0.64]
*AE*	773.16	–114.84	*ACE*	2.30	.130	0.49 [0.36, 0.60]	—	0.51 [0.40, 0.64]
*AE*	773.16	–114.84	*ADE*	0.00	1.000	0.49 [0.36, 0.60]	—	0.51 [0.40, 0.64]
*DE*	778.32	–109.68	*ADE*	5.16	.020	—	0.49 [0.35, 0.60]	0.51 [0.40, 0.65]
***CE***	**771.62**	**–116.38**	***ACE***	**0.76**	**.380**	**—**	**0.42 [0.30, 0.52]**	**0.58 [0.48, 0.70]**
Intraindividual means of negative affect								
*ACE*	–695.16	–1,581.16	—	—	—	0.49 [0.20, 0.62]	0.00 [0.00, 0.21]	0.51 [0.38, 0.67]
*ADE*	–696.06	–1,582.06	—	—	—	0.18 [0.00, 0.61]	0.34 [0.00, 0.64]	0.48 [0.36, 0.65]
*AE*	–695.16	–1,583.16	*ACE*	0.00	1.000	0.49 [0.33, 0.62]	—	0.51 [0.38, 0.67]
*AE*	–695.16	–1,583.16	*ADE*	0.90	.340	0.49 [0.33, 0.62]	—	0.51 [0.38, 0.67]
***DE***	**–695.78**	**–1,583.78**	***ADE***	**0.27**	**.600**	**—**	**0.53 [0.37, 0.65]**	**0.47 [0.35, 0.63]**
*CE*	–687.15	–1,575.15	*ACE*	8.01	.000	—	0.31 [0.18, 0.43]	0.69 [0.57, 0.82]

Note: Values in brackets are 95% confidence intervals. The degrees of freedom for −2 log likelihood (−2LL) is 443 for the full models and 444 for the reduced models. The degrees of freedom for all chi-squared tests is 1. *A* = standardized additive genetic influences; *C* = standardized shared environmental influences; *D* = standardized dominant genetic influences; *E* = standardized nonshared environmental influences; AIC = Akaike’s information criterion. Nonsignificant *p* values indicate that there was no significant deterioration in model fit between the full and the reduced models. The boldface indicates the most parsimonious models for positive affect and negative affect.

**Table 4.** Univariate Model-Fitting Results and Fit Statistics for Intraindividual Standard Deviations of Positive and Negative Affect

			Results of model comparison					
Model	–2LL	AIC	Full model compared	Δχ^2^	*p*	*A*	*C* or *D*	*E*
Intraindividual standard deviations of positive affect								
*ACE*	–432.08	–1,318.08	—	—	—	0.30 [0.06, 0.44]	0.00 [0.00, 0.18]	0.70 [0.56, 0.86]
*ADE*	–434.31	–1,320.31	—	—	—	0.00 [0.00, 0.39]	0.34 [0.00, 0.48]	0.66 [0.52, 0.83]
*AE*	–432.08	–1,320.08	*ACE*	0.00	1.000	0.30 [0.14, 0.44]	—	0.70 [0.56, 0.86]
*AE*	–432.08	–1,320.08	*ADE*	2.23	.130	0.30 [0.14, 0.44]	—	0.70 [0.56, 0.86]
***DE***	**–434.31**	**–1,322.31**	***ADE***	**0.00**	**1.000**	**—**	**0.34 [0.17, 0.48]**	**0.66 [0.52, 0.83]**
*CE*	–427.04	–1,315.04	*ACE*	5.04	.020	—	0.19 [0.06, 0.31]	0.81 [0.69, 0.94]
Intraindividual standard deviations of negative affect								
*ACE*	–277.17	–1,163.17	—	—	—	0.50 [0.28, 0.62]	0.00 [0.00, 0.17]	0.50 [0.38, 0.64]
*ADE*	–279.82	–1,165.82	—	—	—	0.00 [0.00, 0.57]	0.54 [0.00, 0.65]	0.46 [0.35, 0.60]
*AE*	–277.17	–1,165.17	*ACE*	0.00	1.000	0.50 [0.36, 0.62]	—	0.50 [0.38, 0.64]
*AE*	–277.17	–1,165.17	*ADE*	2.65	.100	0.50 [0.36, 0.62]	—	0.50 [0.38, 0.64]
***DE***	**–279.82**	**–1,167.82**	***ADE***	**0.00**	**1.000**	**—**	**0.54 [0.40, 0.65]**	**0.46 [0.35, 0.60]**
*CE*	–265.79	–1,153.79	*ACE*	11.38	.000	—	0.33 [0.20, 0.44]	0.67 [0.56, 0.80]

Note: Values in brackets are 95% confidence intervals. The degrees of freedom for −2 log likelihood (–2LL) is 443 for the full models and 444 for the reduced models. The degrees of freedom for all chi-squared tests is 1. *A* = standardized additive genetic influences; *C* = standardized shared environmental influences; *D* = standardized dominant genetic influences; *E* = standardized nonshared environmental influences; AIC = Akaike’s information criterion. Nonsignificant *p* values indicate that there was no significant deterioration in model fit between the full and the reduced models. The boldface indicates the most parsimonious models for positive affect and negative affect.

According to the most parsimonious models (see the boldface models in Tables 3 and 4), the intraindividual mean of positive affect was the only measure that showed significant shared environmental influences (.42, 95% CI = [.30, .52]) but no heritability. In contrast, the intraindividual mean of negative affect was substantially heritable (.53, 95% CI = [.37, .65]) and without any shared environmental influences. The most parsimonious models for intraindividual variability revealed significant heritability for both negative and positive affect (.54, 95% CI = [.40, .65], and .34, 95% CI = [.17, .48], respectively). Neither measure of intraindividual variability showed any shared environmental influences.

## Discussion

Interindividual differences in the monthlong average of positive affect were not significantly heritable, but rather showed substantial shared environmental influences. By comparison, interindividual differences in the monthlong average of negative affect were substantially heritable, with negligible shared environmental influences, a finding in line with previous reports (Baker et al., 1992; Jacobs et al., 2013; Menne-Lothmann et al., 2012). Our findings support the two-factor model of affect from a genetic perspective. That is, positive affect had primarily a situational etiology and was influenced by environmental experience, whereas negative affect had primarily a dispositional etiology (Diener & Larsen, 1984; Emmons & Diener, 1985). The results suggest that some individuals are genetically driven to feel more or less negative on average than others, whereas individual differences in the average level of positive feelings depend more on shared environmental factors, such as family, school, and neighborhood.

Our study further contributes to the understanding of the genetic architecture of day-to-day intraindividual variability of affect. As did Jacobs et al. (2013), we found that the intraindividual variability of negative and positive affect was significantly heritable. Finding genetic influences on intraindividual variability of affect underlines the limitation of the traditional view of fluctuations in psychological traits and states as merely noise or error (Nesselroade & Ram, 2004; Ram & Gerstorf, 2009; Wang et al., 2012).

What accounts for the heritability of interindividual differences in intraindividual variability of positive and negative affect? One possible explanation involves the norm of reaction (Tabery, 2007), which postulates that individuals with different genotypes demonstrate different ranges within which their phenotypes change in response to different environments. Intraindividual variability of affect could be understood as one indicator of individual lability, or responsivity to the environment and social experience. This interpretation implies that interindividual differences in the extent to which daily mood responds to daily experience and events are heritable. In other words, the potential reaction range of daily mood is heritable. This explanation, which is in line with our findings, demonstrates the dynamic nature of genes in shaping individual characteristics in the face of daily experience, and illustrates the nature of gene-environment interplay. In addition, this explanation is in line with the set-point theory of well-being, which posits that major life events can cause temporal deviations from individuals’ central behavioral tendencies, but that individuals return eventually to their set points (Headey & Wearing, 1992). Finally, our findings are also congruent with the differential-susceptibility theory, which posits that some individuals are more susceptible or malleable to environmental influences than others are, partly because of genetic differences (Belsky, Bakermans-Kranenburg, & van IJzendoorn, 2007).

It is important to emphasize that heritability does not indicate immutability. Therefore, finding substantial heritability for both the intraindividual mean of negative affect and the intraindividual variability of negative affect does not forecast a gloomy prospect for helping people with mood problems or disorders in clinical settings. Heritability only describes the extent to which interindividual differences can be attributed to genetic differences on average in a particular sample at a particular time. In other words, it focuses on group variation rather than group means and on description rather than prediction.

Some limitations of this study should be mentioned. Although it is the largest of its kind regarding both the number of twins in the sample and the number of assessments, our sample was relatively small compared with the samples in conventional twin studies, and our power to detect small effects was limited. Another limitation is our use of the short form of the PANAS, which captures a smaller range of affect states than the full PANAS and as a result may have introduced floor effects that led to the high correlation between intraindividual mean and variability of negative affect. In addition, we did not look at how daily experiences affected participants’ reports of their affect. Future studies could include specific measures of daily events to examine how individuals’ affect changes in the face of particular daily experiences and how these daily events moderate genetic influences on affect in daily life. Also, although our study focused solely on day-to-day fluctuations in affect, momentary fluctuation could be examined with multiple assessments within a day (e.g., Jacobs et al., 2013). Finally, our simple consisted solely of 17-year-olds, and genetic and environmental influences should be examined in other age groups (e.g., adult twins living apart) to determine how these influences change across the life span.

Given the heritability and intraindividual variability of affect, the goal of this study was to examine genetic influences on individual differences in mean affect and day-to-day variability of affect over the course of a month. Personality and cognitive performance have also been shown to be heritable in classical twin designs and to exhibit substantial and meaningful intraindividual variability over time (Baird, Le, & Lucas, 2006; Brose, Schmiedek, Lövdén, & Lindenberger, 2012). One next step would be to investigate the extent to which intraindividual variability of personality and cognitive performance is heritable. Even more informative would be studies of the links between intraindividual variability of psychological processes across domains. For example, Brose et al. (2012) examined the associations between intraindividual variability of cognitive performance and intraindividual variability of affect.

The current study adds to a growing body of behavioral and social science studies that have used intensive longitudinal data to investigate intraindividual variability of various physical- and mental-health outcomes, such as substance use (Shiffman, 2009), attention-deficit/hyperactivity disorder (Rosen & Factor, 2015), and diabetes (Ridenour, Pineo, Molina, & Lich, 2013). For example, intraindividual variability in reaction time (Kuntsi et al., 2013) and intraindividual variability in emotional lability (Merwood et al., 2014) are at the core of attention-deficit/hyperactivity disorder. Unambiguously establishing the heritability of these intraindividual variabilities relevant to a broad range of health outcomes will inform future interventions, such as personalized medicine, so that they can achieve optimal treatment effects.

## Supplementary Material

Supplementary material

## Supplementary Material

Supplementary material

## Notes

1. Recent neuroimaging studies have found no distinct brain areas associated with positive versus negative affect (e.g., Lindquist, Satpute, Wager, Weber, & Barrett, 2016), so although positive and negative affect are typically experienced as distinct states, they are not necessarily encoded by separate brain systems.
