# Supplementary material for: Heritability of Intraindividual Mean and Variability of Positive and Negative Affect: Genetic Analysis of Daily Affect Ratings Over a Month
Source: Psychol Sci. 2016 Oct 11;27(12):1611–9. doi: 10.1177/0956797616669994 (PMC5221725; doi:10.1177/0956797616669994)
Supplement: Supplementary material [file Zheng_Table_S1.doc]

Table S1. *Phenotypic correlations of intra-individual means (iM) and standard deviations (iSD) of positive and negative affect.*

|  | iM_PA | iM_NA | iSD_PA | iSD_NA |
| --- | --- | --- | --- | --- |
| iM_PA | - |  |  |  |
| iM_NA | .00 | - |  |  |
| iSD_PA | .08 | .27^*^ | - |  |
| iSD_NA | .00 | .80^*^ | .43^*^ | - |

^*^ *p* < .001. One twin per pair randomly chosen. PA = positive affect; NA = negative affect.
