# Supplementary material for: Heritability of Intraindividual Mean and Variability of Positive and Negative Affect: Genetic Analysis of Daily Affect Ratings Over a Month
Source: Psychol Sci. 2016 Oct 11;27(12):1611–9. doi: 10.1177/0956797616669994 (PMC5221725; doi:10.1177/0956797616669994)
Supplement: Supplementary material [file Zheng_Table_S2.doc]

*Table S2. Phenotypic correlations of intra-individual means (iM) and standard deviations (iSD) of positive and negative affect among other co-twins*.

|  | iM_PA | iM_NA | iSD_PA | iSD_NA |
| --- | --- | --- | --- | --- |
| iM_PA | - |  |  |  |
| iM_NA | .01 | - |  |  |
| iSD_PA | .09 | .28^*^ | - |  |
| iSD_NA | -.09 | .79^*^ | .47^*^ | - |

^*^ *p* < .001. The other co-twin per pair, non-overlap with those in Table S1. PA = positive affect; NA = negative affect.
